# Successful Treatment of a Granulocytic Sarcoma of the Uterine Cervix in Complete Remission at Six-Year Follow-Up

**DOI:** 10.1155/2010/812424

**Published:** 2010-04-29

**Authors:** Stefano C. H. Kim, Shanti Natarajan-Ame, Bruno Lioure, Marie-Pierre Chenard, Brigitte Duclos, Raoul Herbrecht, Jean-Pierre Bergerat

**Affiliations:** ^1^Department of Hematology and Oncology, Centre des Hôpitaux Universitaires de Strasbourg, BP 67000, Strasbourg, France; ^2^Department of Pathology, Centre des Hôpitaux Universitaires de Strasbourg, Hautepierre 67200, Strasbourg, France

## Abstract

*Background*. Localized granulocytic sarcoma of the uterine cervix in the absence of acute myelogenous leukemia (AML) at presentation is very rare, its diagnosis is often delayed, and its prognosis almost always ominous evolving into refractory AML. *Case*. We present the case of a 30-year-old woman with vaginal bleeding and a large cervical mass. Further evaluation confirmed the presence of a granulocytic sarcoma but failed to reveal systemic involvement. *Results*. AML type chemotherapy followed by radiotherapy of the uterus led to a durable complete remission. She remains in complete remission six years after diagnosis. *Conclusion*. Granulocytic sarcoma of the cervix is a rare entity for which early intensive AML type therapy is effective.

## 1. Introduction

Granulocytic sarcoma (GS) is a rare solid tumor consisting of immature myeloid cells first described as a chloroma by Burns in 1823 because its green color due to myeloperoxidase staining [1, 2]. Its association with acute leukemia was reported by Dock in 1893 [3]. GS is most commonly diagnosed as part of the systemic manifestations of acute myelogenous leukemia (AML), predominantly after the diagnosis of AML, but it may also be the presenting symptom. Less commonly, they signal an AML relapse or blast transformation of chronic myelogenous leukemia (CML). In rare cases, they appear as an isolated mass without prior evidence of leukemia, but in this situation, patients usually develop AML within 8 days to 28 months [4–6]. 

 The incidence of GS is estimated at 0.7 per million in children and 2 per million in adults [7]. It only occurs in 32.3% of patients with granulocytic leukemia, being clinically evident in less than 1% [2]. 

 It most commonly occurs in bones, the periosteum, soft tissues, lymph nodes, and skin but can occur virtually anywhere. The most commonly involved visceral organ is the kidney. 

 GS has a poor prognosis. The overall 2-year survival rate for all patients is 6%. No patient surviving more than 5 years has been reported in the literature [1]. 

 Involvement of the female genital tract in women dying of leukemia is frequent but clinically significant involvement is rare. The most commonly involved organ is the ovary estimated at 36.4% followed by the cervix and uterus [8]. The presenting symptoms of GS of the cervix or uterus include vaginal or postcoital bleeding followed by abdominal pain and systemic manifestations including fever, night sweats, and weight loss [2]. 

 Only a few cases have been reported in which localized GS of the cervix was the only manifestation. Pathak et al. described in their review of 25 patients with GS of the cervix only two cases of confirmed isolated initial cervical involvement. In both cases an AML was subsequently diagnosed. One patient died of the disease, and the other was in remission after acute leukemia chemotherapy in less than 2 years of follow-up [9]. 

 We present here a 30-year-old female with isolated granulocytic sarcoma of the cervix without evolution into AML and in complete remission after 6 years of follow-up.

## 2. Case Report

A 30-year-old, (gravida 1, para 1) white female visited her gynecologist with complaints of irregular vaginal bleeding. Four months earlier, she had had an episode of abnormal vaginal bleeding which lasted two weeks. There was no history of abdominal pain or systemic symptoms. Her last PAP smear, one year prior to diagnosis, was normal. A pelvic examination revealed hypertrophy of the cervix, and cervical biopsy showed an infiltration of small, immature myeloid cell. Laboratory studies were unremarkable. A pelvic CT scan and MRI revealed a mass at the level of the cervix measuring 8 × 5 cm. The mass was homogeneous, well defined, infiltrating, but spared the rectum and the bladder wall. No lymph node enlargement was seen. Ovaries, liver, spleen, pancreas, and kidneys were normal in appearance. There was no ascites and no thoracic involvement. The patient was subsequently referred to our department. 

 She was a social worker with a 2-year-old daughter who had been delivered by cesarean section. The rest of the patient's medical history was unremarkable. 

**Figure 1:**
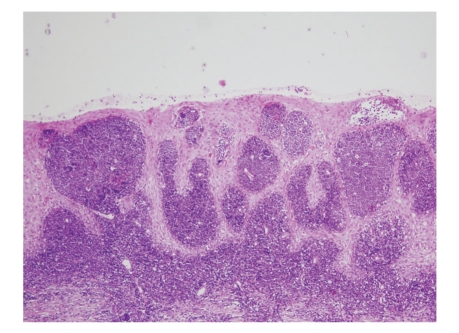
Hematoxylin and eosin stain of granulocytic sarcoma of the uterine cervix.

**Figure 2:**
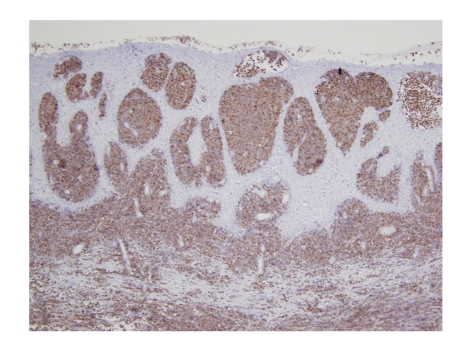
Myeloperoxidase stain of granulocytic sarcoma of the uterine cervix.

 On examination, she was in perfect physical condition, no history of weight loss, fever, or night sweats, and the blood pressure was 110/70 mm Hg. Gynecologic examination revealed an enlarged cervix, which was firm, elastic and bruised easily without parametrial or vaginal cul-de-sac involvement. There were no palpable lymph nodes and no hepatosplenomegaly. The rest of the physical examination was normal. Laboratory studies revealed the following values: hemoglobin 13.3 g/dL, platelet 218 × 10^3^/mL, and white-cell count 7300 per cubic millimeter, with 62.5% neutrophils, 30.2% lymphocytes, and 5.3% monocytes. Blood chemistry was unremarkable. Cytological examination of cervical smears was composed of 50% of hematopoietic cells, small to medium sized blasts, with high nuclears-cytoplasmic ratio, fine chromatin, and fine granulations in the cytoplasm of myeloid origin. The blasts were myeloperoxidase positive. A biopsy was performed (two fragments of 1.3 × 0.5 × 0.2 and 1.1 × 0.7 × 0.2 cm) which showed extensive infiltration of the squamous epithelium wall by small-sized blast cells. Multiple immunohistochemical analyses were obtained. Myeloperoxidase, Common leucocytic antigen, and CD34 were strongly positive. (Figures 1 and 2) Lymphoid markers, epithelial markers, and neuroendocrine markers were all negative (CD20, CD3, CD23, CD5, CD10, CD68, CD45 RO, Kappa, Lambda, cytokeratin, EMA, synaptophysin, and chromogranin). The Ki67 was expressed in 80% of the cells. These findings were compatible with the diagnosis of a GS of the myeloblastic type. Bone marrow biopsy (BMB) and aspirate were normal as were cytogenetics. 

 As most patients with a localized GS evolve into an AML, our patient received AML type induction chemotherapy with cytosine arabinoside (200 mg/m²/day for seven days) and idarubicin (8 mg/m²/day for five days). A postchemotherapy examination of the cervix was normal, and a biopsy performed one month after chemotherapy confirmed the absence of residual blast cells. The patient desired subsequent pregnancies in the future and so the following treatment strategy was adopted: one cure of consolidation chemotherapy with high-dose cytosine arabinoside (3 g/m² × 2/day for 4 days) and idarubicin (12 mg/m²/day for 2 days) followed by laparoscopic protective ovariopexy and external beam radiotherapy of 30 Gy limited to uterus. This was followed by a second cycle of consolidation chemotherapy with high dose cytosine arabinoside (3 g/m² × 2/day for 4 days).

 At the last follow-up in December 2008, more than six years after the diagnosis, she remains in complete remission. Complete blood counts, abdominal and pelvic ultrasonography, and physical examination were normal.

## 3. Discussion

The mean age at presentation of cervical granulocytic sarcoma is 47 years, range from 26 to 75 of age [1]. The two patients with isolated GS of the uterine cervix at diagnosis described by Pathak et al. were 32 and 34 years of age, similar to our patient. 

 The presenting feature in our case was vaginal bleeding as with the majority of patients: 81–83% of presenting symptoms. Abdominal pain or discomfort present in 17–29% of patients and systemic symptoms in 6–17% were absent in our patient. Finally a combination of other symptoms is responsible for 17% of initial complaints [1, 2]. 

 The diagnosis is not always easy when GS appears at an extramedullary site in a nonleukemic patient. Most of them are poorly differentiated, and only in 44% of cases the correct diagnosis is made or suspected [9, 10]. The most common misdiagnosis is the high grade non-Hodgkin lymphoma. Both are composed of diffusely infiltrating, discohesive cells that tend to spare normal structures, and which may contain scattered lymphocytes. In GS, however, the nuclei are typically slightly smaller with more finely dispersed chromatin, and some cell may show recognizable myeloid differentiation. The immunohistochemical stains are usually diagnostic [8]. 

 GS of the cervix has a poor prognosis. Treatment is variable but often delayed. Few reports of complete remission have been described after aggressive multimodality treatment of GS in other sites including disseminated GS without evidence of AML [11]. 

 Our patient was treated with AML type chemotherapy regimen and localized uterine radiotherapy. A complete response was seen soon after treatment, and she remains in complete remission. It appears appropriate to treat GS with AML type chemotherapy protocols even in the absence of systemic manifestations, since acute myeloid leukemia will almost always be present [10]. To our knowledge this is the first case of an isolated GS of the uterine cervix without progression into AML. 

 In the hope of maintaining fertility, she was treated with an LHRH analogue during chemotherapy. She had an ovariopexy before radiotherapy and recovered her menstrual cycles seven months after treatment. Unfortunately she was unable to conceive and even failed several attempts at in vitro fertilization. 

 Our patient received a total radiation dose of 32.4 Gy. The irradiated uterus suffers somatic damage; it causes endometrial and myometrial atrophy, scar fibrosis, and hypovascularization which interfere with implantation [12]. Independently of other factors, irradiation doses of greater than 30 Gy appear to be determinant for fertility. Nevertheless some cases of pregnancy following uterine irradiation of up to 30 Gy have been reported in the literature [13].

## 4. Conclusion

Granulocytic sarcoma of the cervix is a rare entity and even in its localized form, early intensive AML type therapy appears to be appropriate. Even though our patient has had a long complete remission, the role of radiotherapy in addition to chemotherapy remains unclear.

##  Conflict of Interest

The authors declare that there are no conflicts of interest.
